# Draft Genome Sequence of *Pseudoalteromonas* sp. Strain ND6B, an Oil-Degrading Isolate from Eastern Mediterranean Sea Water Collected at a Depth of 1,210 Meters

**DOI:** 10.1128/genomeA.01212-14

**Published:** 2014-11-26

**Authors:** Austin P. Harris, Stephen M. Techtmann, Savannah C. Stelling, Sagar M. Utturkar, Noor K. Alshibli, Steven D. Brown, Terry C. Hazen

**Affiliations:** a Department of Civil and Environmental Engineering, University of Tennessee, Knoxville, Tennessee, USA; b Center for Environmental Biotechnology, University of Tennessee, Knoxville, Tennessee, USA; c Biosciences Division, Oak Ridge National Laboratory, Oak Ridge, Tennessee, USA; d Graduate School of Genome Science and Technology, University of Tennessee, Knoxville, Tennessee, USA

## Abstract

Here, we report the draft genome of *Pseudoalteromonas* sp. strain ND6B, which is able to grow with crude oil as a carbon source. Strain ND6B was isolated from eastern Mediterranean Sea deep water at a depth of 1,210 m. The genome of strain ND6B provides insight into the oil-degrading ability of the *Pseudoalteromonas* species.

## GENOME ANNOUNCEMENT

Marine *Gammaproteobacteria* of the genus *Pseudoalteromonas* have been isolated from locations around the globe under many different environmental conditions. Over 40 draft genomes for *Pseudoalteromonas* species are currently available. *Pseudoalteromonas* are increasingly recognized as ecologically and biotechnologically important microbes. Several genera within the *Gammaproteobacteria* have evolved the ability to degrade many of the components of petroleum (1, 2). Some of these hydrocarbon-degrading *Pseudoalteromonas* strains were important members of the microbial community that responded to the Deepwater Horizon oil spill (3, 4).

*Pseudoalteromonas* sp. strain ND6B was isolated from water at a depth 1,210 m in the eastern Mediterranean Sea (29.571°E, 31.813°N). *Pseudoalteromonas* sp. strain ND6B was isolated on ONR7a medium (5) supplemented with 100 ppm of Norne Blend crude oil as the carbon source. Analysis of the 16S rRNA gene sequence indicated that *Pseudoalteromonas* sp. ND6B is most closely related to *Pseudoalteromonas* sp. SM9913 (99% 16S rRNA gene identity). *Pseudoalteromonas* sp. SM9913 was isolated from sediments in the Okinawa Trough and has been proposed as a model organism for deep-sea heterotrophy (6). Environmental conditions and nutrient fluxes in the Okinawa Trough are distinct from the eastern Mediterranean Sea water column. The eastern Mediterranean Sea is characterized by high salinity, low nutrient concentrations, and elevated bottom water temperatures (13.8°C) (7, 8). To better understand deep-sea *Pseudoalteromonas* species, the genome of *Pseudoalteromonas* sp. ND6B was sequenced.

Draft genome sequence for *Pseudoalteromonas* sp. strain ND6B was generated using the Illumina MiSeq platform, which generated 6,381,754 paired-end reads. Quality-based trimming was performed using Trimmomatic with the following parameters: SLIDINGWINDOW:4:15, MINLEN:36 (9). After quality filtering, 5,576,996 paired-end reads remained, resulting in 2,018,633,142 bp of sequence data with an average read length of 198 bp. After testing several approaches (10), the genome was assembled using ABySS (11) into 90 large (≥500 bp) contigs, with a total genome size of 4.2 Mb. The *N*_50_ contig size was 136,207 bp, with the largest contig being 330,336 bp. Genes were identified using the Prodigal algorithm (12) as part of the Oak Ridge National Laboratory genome annotation pipeline.

The draft genome has an overall G+C content of 40.3% and 3,798 candidate protein-encoding genes. Putative functions from functional clusters of orthologous groups (COG) were assigned to 76.3% of the candidate genes. Strain ND6B contains 20 monooxygenases and dioxygenases including a phenol monooxygenase gene, which is important for degradation of various phenolic compounds. The presence of these predicted oxygenase genes in part explains the ability of strain ND6B to use oil as a carbon source. Furthermore, strain ND6B encodes multiple homologs of the secreted metalloproteases related to MCP-02 and MCP-03 identified in *Pseudoalteromonas* sp. SM9913 (13). These metalloproteases were proposed to be important for sedimentary nitrogen degradation. The presence of these proteases in strain ND6B, which was isolated from the water column, suggests a more generic role for these proteases in deep-sea heterotrophic growth and nitrogen degradation in the deep ocean.

### Nucleotide sequence accession number.

The draft genome sequence of strain ND6B has been deposited at DDBJ/EMBL/GenBank under the accession no. JQFL00000000. The version described in this paper is the first version.
